# Impact of Insall-Salvati index on clinical outcomes after medial patellofemoral ligament reconstruction in children: a retrospective single-center study

**DOI:** 10.3389/fped.2026.1887245

**Published:** 2026-10-28

**Authors:** Chi Zhang, Shaosong Sun, Xingchen Zhang, Bin Deng, Shengcheng Yao

**Affiliations:** Department of Orthopedics, Affiliated Hospital of Xuzhou Medical University, Xuzhou, Jiangsu, China

**Keywords:** child, joint instability, patella, patellar dislocation, reconstructive surgical procedures, treatment outcome

## Abstract

**Background:**

Patella alta is a recognized risk factor for patellar instability, yet its influence on outcomes after medial patellofemoral ligament (MPFL) reconstruction in pediatric patients remains poorly defined. This study evaluated the association between preoperative patellar height, measured by the Insall-Salvati index (ISI), and clinical outcomes following MPFL reconstruction in children with recurrent patellar instability, with the Caton-Deschamps index (CDI) assessed as a comparator measure of patellar height.

**Methods:**

This retrospective single-center study included pediatric patients (<18 years) who underwent MPFL reconstruction for recurrent lateral patellar dislocation between January 2022 and April 2025, with a median follow-up of 29.9 months (range 9–51 months). Patients were stratified into a patella alta group (ISI > 1.2) and a normal patellar height group (ISI ≤ 1.2). The primary outcome was the Kujala score at final follow-up. Secondary outcomes included the Lysholm score, redislocation and subluxation rates, and ISI change. Correlations between preoperative ISI and functional outcomes were analyzed, and multivariable regression models were used to determine whether preoperative ISI was independently associated with outcomes after adjustment for potential confounders.

**Results:**

Of 103 patients, 49 (47.6%) were classified as patella alta. Both groups demonstrated significant postoperative ISI reductions, with a greater decrease observed in the patella alta group. At final follow-up, the patella alta group had significantly lower Kujala and Lysholm scores, and higher rates of redislocation, subluxation, and overall poor outcomes compared with the normal group. Preoperative ISI correlated negatively with the final Kujala score (*ρ* = −0.457, *P* < 0.001), whereas the Caton-Deschamps index (CDI) showed no significant correlation. After multivariable adjustment, each 0.1-unit increase in preoperative ISI remained associated with a poor outcome (adjusted odds ratio 1.36, *P* = 0.040) and with a lower final Kujala score (*P* < 0.001). Postoperative ISI normalization, achieved in 24 of 49 patients (49.0%) in the patella alta group, was not associated with improved clinical outcomes.

**Conclusion:**

Preoperative patella alta, as defined by an elevated ISI, was associated with inferior functional and stability outcomes after MPFL reconstruction in children. In this cohort, the ISI was more strongly associated with clinical outcomes than the CDI, and this association persisted after multivariable adjustment; however, given the retrospective observational design, these findings should be regarded as hypothesis-generating and warrant confirmation in larger prospective studies. These findings suggest that preoperative ISI assessment may be valuable for risk stratification and patient counseling when planning isolated MPFL reconstruction in children.

## 1 Introduction

Lateral patellar dislocation is among the most common acute knee injuries in the pediatric and adolescent population, with a cumulative incidence peaking at 147.7 per 100,000 person-years in the 14–18-year age group (1). Epidemiological data have demonstrated a significant upward trend in both the frequency of acute patellar dislocation events presenting to emergency departments and the rate of surgical procedures for patellar instability in this age group over the past two decades (2], (3). Following a primary dislocation, the reported rate of recurrence ranges from 17% to 66% (4). Recurrent episodes are associated with cartilage damage, anterior knee pain, and long-term functional impairment, which collectively underscore the need for effective surgical intervention in selected patients (5).

Medial patellofemoral ligament (MPFL) reconstruction has emerged as the preferred surgical treatment for recurrent patellar instability in both adults and children, although it encompasses a range of surgical techniques, fixation methods, and graft configurations rather than a single standardized procedure (6, 7). In the pediatric setting, MPFL reconstruction has been shown to yield favorable functional outcomes, with postoperative Kujala scores typically exceeding 85 points and redislocation rates of approximately 5% to 10% at short- to mid-term follow-up (8, 9). Although surgical techniques have advanced, treatment failures still persist, and preoperative risk factors that compromise outcomes remain under investigation. Various risk factors, including trochlear dysplasia, femoral tunnel malposition, and patella alta, have been associated with worse outcomes after MPFL reconstruction (10).

Patella alta, defined as an abnormally proximal position of the patella relative to the femoral trochlea, is a well-established pathoanatomic contributor to patellar instability (11). A high-riding patella delays engagement with the trochlear groove during early knee flexion, thereby reducing the bony constraint on the patella in the range where the MPFL serves as the primary soft-tissue stabilizer (5, 11). Patellar height has likewise been shown to influence patellofemoral stability in other surgical settings, underscoring its broader role as an anatomical determinant of knee stability (12). The Insall-Salvati index (ISI), calculated as the ratio of patellar tendon length to patellar diagonal length on lateral radiographs, is one of the earliest and most widely used methods for quantifying patellar height (13). Each index has distinct strengths and limitations: the CDI references the patellar articular surface and the anterosuperior tibial plateau and is therefore less susceptible to variation in patellar morphology and tendon attachment, whereas the ISI relies on the patellar tendon and the patellar diagonal length, landmarks that remain clearly identifiable even when the articular surface is incompletely ossified in younger children (13, 14). The clinical relevance of patellar height assessment using these indices has been demonstrated across a range of knee conditions (15, 16). In a study of 211 isolated MPFL reconstructions, patella alta measured by a CDI index of 1.3 or greater was identified as an independent predictor of surgical failure, with an odds ratio of 4.9 (17). Similarly, patients with elevated patellar height who underwent isolated MPFL reconstruction have been reported to experience significantly higher rates of postoperative instability and revision surgery compared to those with normal patellar height (18). Importantly, the MPFL reconstruction studies discussed above are based predominantly on adult or mixed-age cohorts, and the specific role of patellar height in determining outcomes of MPFL reconstruction in a purely pediatric population remains unclear; dedicated pediatric evidence is therefore needed. This knowledge gap is particularly relevant in the skeletally immature population for several reasons. Because the pediatric knee is still growing, patellar height indices that depend on incompletely ossified landmarks may behave differently than in adults, and the choice of measurement index may itself influence prognostic conclusions. Skeletal immaturity also constrains the surgical options, mandating physeal-sparing reconstruction and precluding the concurrent bony realignment procedures frequently used in adults to address patella alta. Moreover, adolescents carry the highest risk of recurrent patellar instability, so identifying preoperative anatomical predictors that remain informative in this specific age group has direct clinical value. Evidence extrapolated from adult or mixed-age cohorts may therefore not be directly applicable to children, underscoring the need for dedicated pediatric data. Against this background, the ISI was prespecified as the primary patellar height index in the present study because its reference landmarks remain reliably measurable throughout skeletal growth, whereas the articular-surface landmarks on which the CDI depends undergo morphological change during ossification; the CDI was nevertheless evaluated in parallel to permit a direct, within-cohort comparison of the two indices (19, 20). It has also been observed that the Insall-Salvati index did not decrease significantly after isolated MPFL reconstruction in patients with patella alta, in contrast to the CDI index, raising the question of whether these two indices capture different prognostic information (14).

The aim of this study was to evaluate the influence of preoperative patellar height, as measured by the ISI, on clinical and radiographic outcomes following MPFL reconstruction in pediatric patients with recurrent patellar instability, and to compare the prognostic performance of the ISI with that of the CDI.

## 2 Methods

### 2.1 Study design

This retrospective, single-center, comparative study was conducted at Affiliated Hospital of Xuzhou Medical University and included pediatric patients who underwent MPFL reconstruction for recurrent lateral patellar dislocation between January 2022 and April 2025. The study protocol was approved by the Institutional Review Board of Affiliated Hospital of Xuzhou Medical University (approval number:XYFY2025-KL687-01), and the requirement for written informed consent was waived given the retrospective nature of the data analysis. This report adheres to the Strengthening the Reporting of Observational Studies in Epidemiology (STROBE) guidelines (21).

### 2.2 Patient screening and grouping

Patients were eligible if they fulfilled all of the following criteria: 1) clinical diagnosis of recurrent lateral patellar dislocation with at least two documented episodes; 2) younger than 18 years at the time of surgery; 3) underwent MPFL reconstruction during the enrollment period; 4) available lateral knee radiographs from preoperative and postoperative periods; 5) minimum postoperative follow-up of 6 months. Patients were excluded if any of the following applied: concomitant tibial tubercle transfer, trochleoplasty, or corrective osteotomy performed at the index procedure; concurrent cruciate ligament injury or meniscal pathology requiring surgical treatment; syndromic or congenital patellar dislocation; habitual patellar dislocation defined as obligatory dislocation during active knee flexion; previous surgery on the ipsilateral knee; or incomplete clinical or radiographic documentation.

Patients were stratified into two groups according to the preoperative ISI: the patella alta group (ISI > 1.2) and the normal patellar height group (ISI ≤ 1.2) (13, 22). This threshold was chosen because an ISI of approximately 1.2 corresponds to the upper limit of the normal range reported in both adult and pediatric cohorts, and because the Insall-Salvati ratio—being based on the patellar tendon-to-patella length ratio rather than on articular-surface landmarks—is comparatively robust to the incomplete epiphyseal ossification of the growing knee, making it particularly suitable for a pediatric population (13, 19).

### 2.3 Surgical technique

All procedures were performed by the same surgical team using a uniform MPFL reconstruction technique. The graft consisted of a hamstring tendon autograft (gracilis or semitendinosus) harvested from the ipsilateral limb. On the patellar side, fixation was achieved through two bone tunnels drilled in the superomedial border of the patella, supplemented by suture anchors. On the femoral side, the anatomic insertion site of the MPFL was located under fluoroscopic guidance using the radiographic landmarks (23). In skeletally immature patients, an entirely epiphyseal, physeal-sparing technique was used. The femoral fixation point was placed within the distal femoral epiphysis, distal to the distal femoral physis, and neither the tunnel nor the fixation device crossed the growth plate; the epiphyseal position and its relationship to the physis were confirmed under fluoroscopy to avoid iatrogenic physeal injury. The graft was fixed within the epiphysis using an interference screw, supplemented by a suture anchor when additional fixation was required. Graft tensioning was performed with the knee held at 30 degrees of flexion to reproduce the native isometric behavior of the MPFL, and femoral fixation was completed with an interference screw or anchor.

Postoperative rehabilitation followed a standardized protocol. A hinged knee brace locked in full extension was worn for the initial four weeks. Range of motion exercises were initiated at 2 weeks postoperatively and progressed gradually. Partial weight-bearing was allowed immediately, and full weight-bearing was permitted at 4 to 6 weeks. Non-contact sporting activities were authorized at 3 months, while unrestricted return to full athletic participation required a minimum of 6 months.

### 2.4 Data collection

1. Baseline demographic and clinical data were extracted from the medical records, including age at surgery, sex, body mass index (BMI), affected side (left, right, or bilateral), age at the initial dislocation event, total number of preoperative dislocations, time interval from the most recent dislocation to surgery, history of contralateral patellar instability, family history, and generalized joint hypermobility assessed by the Beighton score (24).
2. All radiographic data were obtained through retrospective review of archived imaging records in the picture archiving and communication system. Radiographic parameters were measured on standard true lateral knee radiographs obtained at 30 degrees of flexion, both preoperatively and at the most recent follow-up. The ISI, defined as the ratio of patellar tendon length to the greatest diagonal length of the patella on lateral radiographs, was the primary imaging variable. The CDI was defined as the ratio of the distance between the inferior articular margin of the patella and the anterosuperior angle of the tibial plateau to the length of the patellar articular surface (25). The tibial tubercle to trochlear groove (TT-TG) distance was measured on axial magnetic resonance imaging (MRI) (26). Trochlear morphology was classified into Dejour types A through D (27). The patellar tilt angle (28), sulcus angle (29), and lateral trochlear inclination (30) were additionally measured on the same axial MRI sequences. All radiographic and MRI measurements were performed independently by two fellowship-trained musculoskeletal physicians. The mean of the two values was used for analysis, and interobserver reliability was quantified using the intraclass correlation coefficient (two-way random-effects model, absolute agreement).
3. Functional outcome assessments were conducted preoperatively and at the final follow-up visit. The Kujala anterior knee pain score (range 0 to 100, with higher scores indicating better function) and the Lysholm knee score (range 0 to 100) were recorded (31, 32). Knee range of motion (ROM) and a visual analog scale (VAS) pain score (0 to 10) were also documented at both time points. Additional clinical outcomes recorded at the final follow-up included the occurrence of patellar redislocation or subluxation, a positive apprehension test, complications (wound infection, arthrofibrosis, and hardware-related problems), and the timing of return to sporting activities.

### 2.5 Definition of outcomes

The primary outcome was the Kujala score at the final postoperative follow-up. The Kujala score was designated as the primary outcome because it is a disease-specific instrument developed and validated for patellofemoral disorders and is therefore more sensitive to the functional limitations most relevant to this population than generic knee scores; prespecifying a single primary outcome also serves to limit multiplicity in hypothesis testing. The remaining outcomes were classified as secondary because they provide supportive and contextual information on symptoms, stability, and radiographic change. Secondary outcomes included the Lysholm score, the rate of redislocation or subluxation, and the magnitude of ISI change from the preoperative to the postoperative measurement. A poor outcome was defined as the occurrence of at least one episode of patellar redislocation or subluxation during the follow-up period.

### 2.6 Statistical analysis

Continuous data were assessed for normality using the Shapiro–Wilk test. Variables that followed a normal distribution are presented as mean ± standard deviation (SD), and between-group comparisons were performed using the independent *t*-test. Variables that did not follow a normal distribution are presented as median with interquartile range (IQR), and between-group comparisons were performed using the Mann–Whitney U test. Categorical data are presented as *n* (%) and were compared using the chi-square test or the Fisher exact test when any expected cell count was below 5. Paired comparisons of preoperative and postoperative measurements within each group were performed using the paired-samples *t*-test for normally distributed data or the Wilcoxon signed-rank test for non-normally distributed data. Correlations between the preoperative patellar height indices (ISI and CDI) and the postoperative functional scores were assessed using the Pearson correlation coefficient for normally distributed data or the Spearman rank correlation coefficient for non-normally distributed data, and the agreement between the ISI and the CDI in classifying patellar height was evaluated using the Cohen kappa coefficient. To determine whether preoperative ISI was independently associated with clinical outcomes, multivariable logistic regression (for the occurrence of a poor outcome) and multivariable linear regression (for the final Kujala score) were performed, with adjustment for BMI, preoperative Lysholm score, high-grade trochlear dysplasia (Dejour type B or D), and TT-TG distance. The associations of preoperative and postoperative ISI with recurrent instability were additionally examined using logistic regression. For each patient, the number of coexisting anatomical risk factors (patella alta, high-grade trochlear dysplasia, TT-TG ≥ 20 mm, and patellar tilt > 20°) was counted, and the relationship between the number of risk factors and poor outcome was assessed using the Cochran-Armitage test for trend. Prespecified subgroup analyses were performed according to chronological age (≤ 15 vs. > 15 years) and skeletal maturity (open vs. closed distal femoral physis), with effect modification evaluated using multiplicative interaction terms.

Patients with missing data for any variable were excluded from the corresponding analysis on a complete-case basis, and the proportion of missing data for each variable was reported. All analyses were performed using SPSS version 26.0 (IBM Corp., Armonk, NY, USA). Statistical significance was set at a two-tailed *P* < 0.05.

## 3 Results

### 3.1 Patient demographics and baseline characteristics

A total of 156 patients who underwent MPFL reconstruction during the study period were screened, of whom 53 were excluded based on predefined criteria (Figure 1).

**Figure 1:**
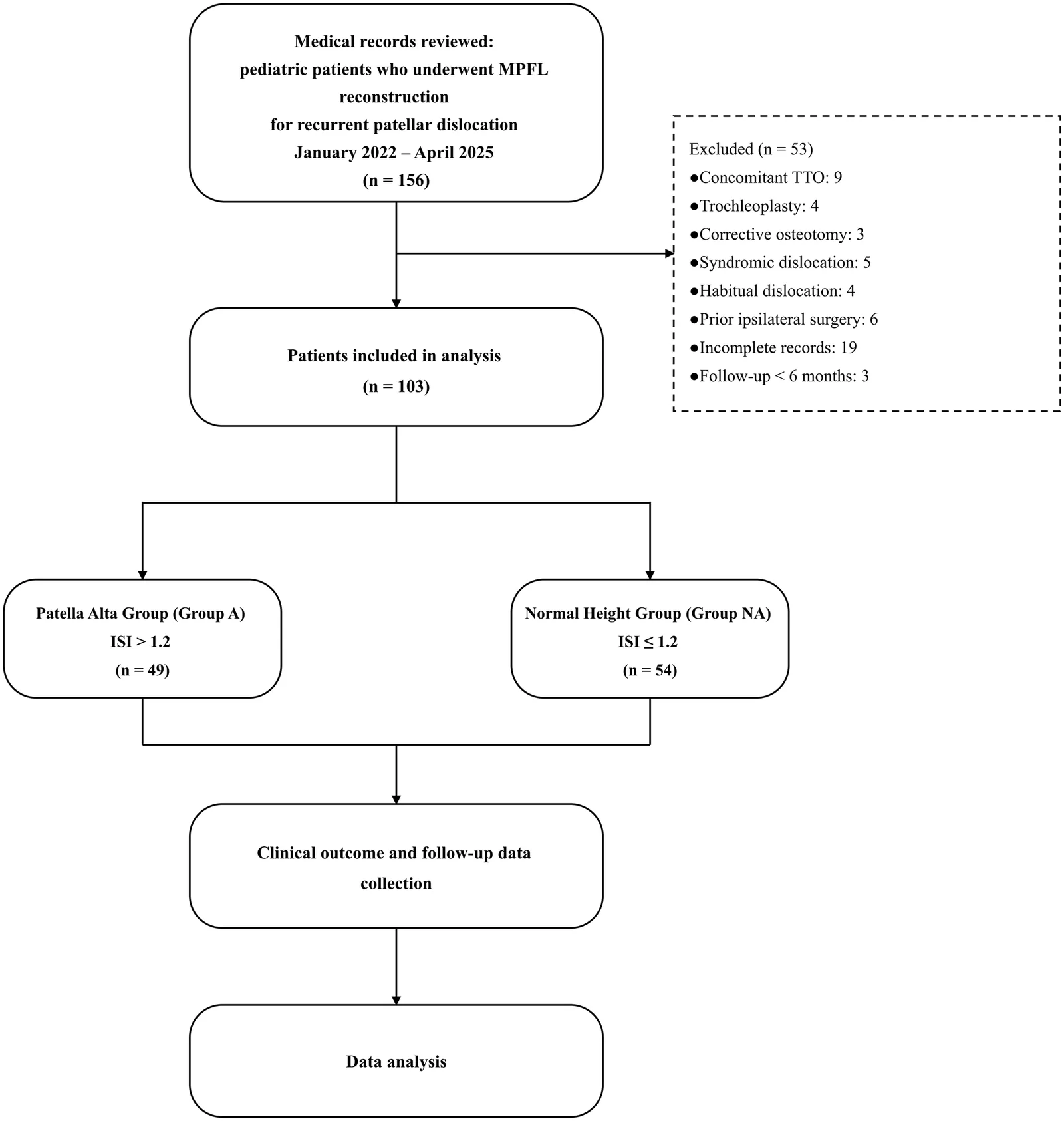
STROBE flow diagram. ISI, Insall-Salvati index; MPFL, medial patellofemoral ligament. Group A denotes the patella alta group (ISI > 1.2), and Group NA denotes the group without patella alta (normal patellar height, ISI ≤ 1.2).

The final cohort comprised 103 patients (65 female, 38 male) with a mean age of 13.7 ± 2.2 years (range 8–18). The median follow-up was 29.9 months (range 9–51). Based on preoperative ISI, 49 patients (47.6%) were classified into the patella alta group (ISI > 1.2) and 54 patients (52.4%) into the normal patellar height group (ISI ≤ 1.2).

The detail demographics and baseline characteristics are shown in Table 1. The two groups were comparable in age, sex, Beighton score, TT-TG distance, trochlear dysplasia distribution, preoperative J-sign, skeletal maturity status, number of prior dislocations, and the preoperative Kujala score (all *P* > 0.05). BMI was slightly higher in the patella alta group (*P* = 0.047). In addition, the preoperative Lysholm score was lower (*P* = 0.043), and the interval from the most recent dislocation to surgery was shorter (*P* = 0.013), in the patella alta group. These baseline differences were subsequently accounted for in the multivariable analysis (Section 3.5). The interobserver reliability of the radiographic and MRI measurements was good, with an overall intraclass correlation coefficient of 0.893.

**Table 1:** Baseline demographics and preoperative characteristics.

Variable	Patella Alta (*n* = 49)	Normal (*n* = 54)	*P* value
Demographics			
Age at surgery (years)	13.9 ± 2.2	13.5 ± 2.2	0.349^a^
Sex, *n* (%)			0.863^c^
Male	19 (38.8)	19 (35.2)	
Female	30 (61.2)	35 (64.8)	
BMI (kg/m²)	19.2 (17.2–23.6)	18.0 (16.2–21.4)	0.047^b^
Open physis, *n* (%)	31 (63.3)	36 (66.7)	0.877^c^
Affected side, *n* (%)			
Left	25 (51.0)	17 (31.5)	
Right	17 (34.7)	32 (59.3)	
Bilateral	7 (14.3)	5 (9.3)	
Clinical history			
Age at first dislocation (years)	11.0 ± 2.8	11.3 ± 2.7	0.573^a^
Number of dislocations	3.0 (2.0–4.0)	2.0 (2.0–3.0)	0.052^b^
Time from last dislocation to surgery (months)	1.8 (1.2–4.6)	4.0 (2.2–5.7)	0.013^b^
Beighton score (0–9)	4.2 ± 2.0	3.9 ± 2.0	0.544^a^
Generalized joint hypermobility, *n* (%)	32 (65.3)	32 (59.3)	0.668^c^
Contralateral instability, *n* (%)	11 (22.4)	14 (25.9)	0.856^c^
Family history, *n* (%)	14 (28.6)	11 (20.4)	0.460^c^
Preoperative imaging			
Insall-Salvati index	1.3 (1.2–1.4)	1.1 (1.0–1.1)	<0.001^b^
Caton-Deschamps index	1.2 ± 0.2	1.1 ± 0.2	0.205^a^
Blackburne-Peel ratio	0.9 ± 0.2	0.9 ± 0.2	0.465^a^
TT-TG distance (millimeters)	17.3 ± 3.6	17.1 ± 4.5	0.805^a^
Patellar tilt angle (degrees)	19.6 ± 6.3	17.9 ± 6.2	0.163^a^
Sulcus angle (degrees)	151.2 ± 8.1	151.3 ± 7.2	0.907^a^
Lateral trochlear inclination (degrees)	11.4 ± 5.1	11.6 ± 4.7	0.874^a^
Trochlear dysplasia (Dejour), *n* (%)			
Type Normal	6 (12.2)	7 (13.0)	
Type A	10 (20.4)	12 (22.2)	
Type B	9 (18.4)	10 (18.5)	
Type C	14 (28.6)	14 (25.9)	
Type D	10 (20.4)	11 (20.4)	
Trochlear bump present, *n* (%)	19 (38.8)	21 (38.9)	1.000^c^
Preoperative clinical examination			
Positive J-sign, *n* (%)	35 (71.4)	38 (70.4)	1.000^c^
Positive apprehension test, *n* (%)	45 (91.8)	49 (90.7)	1.000^d^
Preoperative functional scores			
Kujala score (0–100)	51.3 ± 10.8	55.1 ± 12.7	0.107^a^
Lysholm score (0–100)	57.7 ± 11.4	62.3 ± 11.6	0.043^a^
VAS pain score (0–10)	5.2 ± 1.6	5.3 ± 1.8	0.659^a^
Follow-up duration (months)	27.5 (17.2–38.9)	33.0 (18.8–39.7)	0.452^b^

Data are presented as mean ± SD for normally distributed continuous variables, median (Q1, Q3) for non-normally distributed continuous variables, and *n* (%) for categorical variables. Normality was assessed using the Shapiro–Wilk test. ISI, Insall-Salvati index; CDI, Caton-Deschamps index; TT-TG, tibial tubercle-trochlear groove distance; BMI, body mass index; ROM, range of motion; VAS, visual analog scale.

a Idependent samples t-test.

b Mann–Whitney U test.

c Chi-square test.

d Fisher's exact test.

### 3.2 Changes in patellar height after MPFL reconstruction

Both groups demonstrated postoperative reductions in ISI (both *P* < 0.001) (Figure 2A). In the patella alta group, the mean ISI decreased from 1.301 to 1.223, yielding a mean change of 0.077. In the normal group, the mean ISI decreased from 1.060 to 1.033, with a mean change of 0.027. The magnitude of ISI reduction was significantly greater in the patella alta group (*P* < 0.001) (Figure 2B). Among the 49 patella alta patients, 24 (49.0%) achieved postoperative ISI normalization (≤1.2).

**Figure 2:**
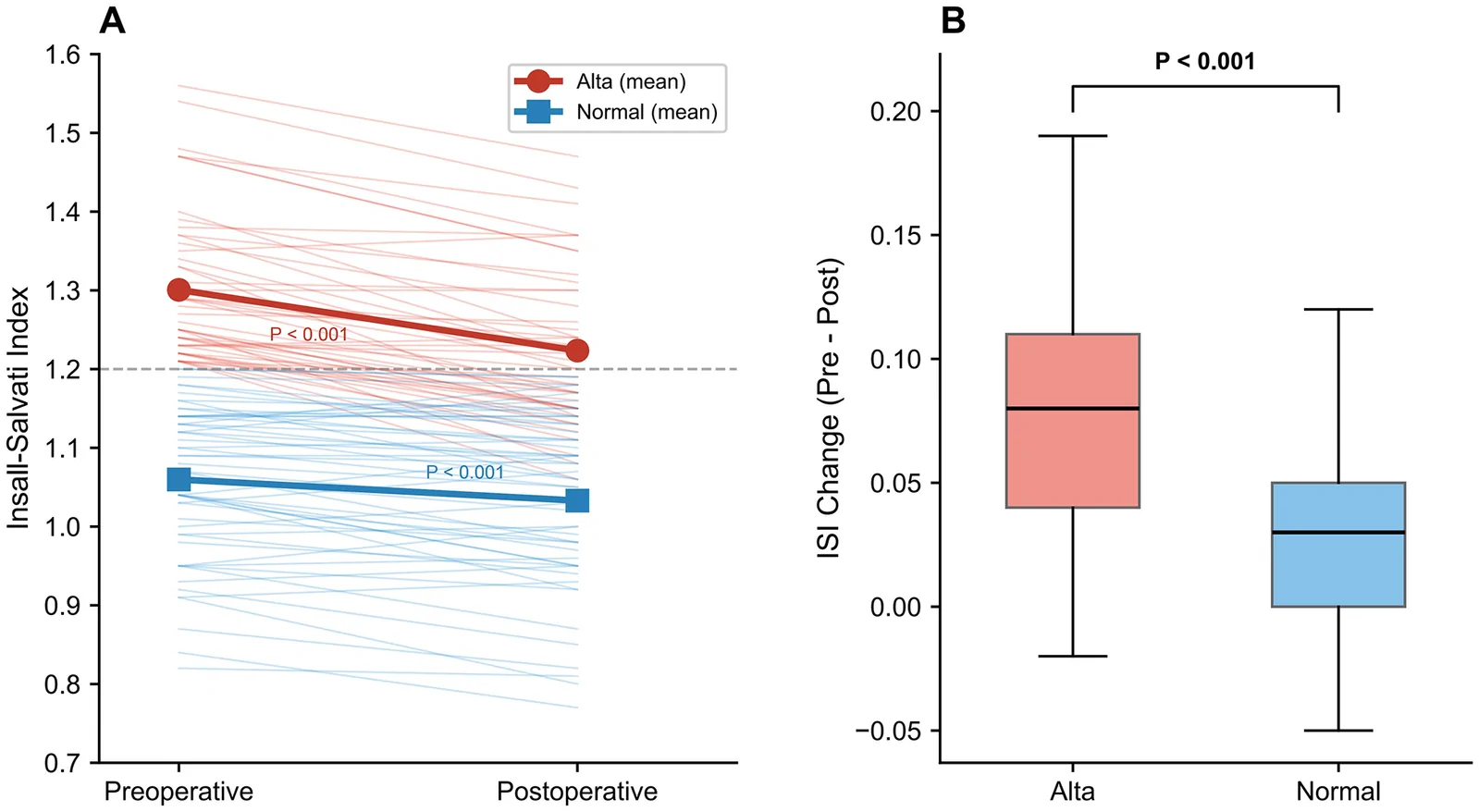
Changes in Insall-Salvati index after MPFL reconstruction. **(A)** Individual patient trajectories from preoperative to postoperative ISI values. The dashed horizontal line marks the ISI = 1.2 threshold; **(B)** Box plot comparing ISI change magnitude (preoperative minus postoperative) between the two groups. ISI, Insall-Salvati index; MPFL, medial patellofemoral ligament.

CDI also decreased significantly in both groups after surgery (both *P* < 0.001); however, the between-group difference in CDI change did not reach statistical significance (*P* = 0.869). The agreement between ISI and CDI in classifying patellar height was poor (Cohen's kappa = −0.017). Of the 49 patients classified as patella alta by ISI, only 11 (22.4%) were simultaneously classified as patella alta by CDI.

### 3.3 Clinical outcomes

Postoperative clinical outcomes are summarized in Table 2. Both groups showed significant improvement in Kujala score, Lysholm score, and VAS from preoperative to final follow-up (all within-group *P* < 0.001). At final follow-up, the patella alta group had lower Kujala scores (*P* < 0.001) and Lysholm scores (*P* = 0.003) compared to the normal group. No significant between-group difference was observed in postoperative VAS. Postoperative range of motion was lower in the patella alta group (*P* < 0.001).

**Table 2:** Postoperative clinical outcomes of two groups.

Variable	Patella Alta (*n* = 49)	Normal (*n* = 54)	*P v*alue
Postoperative patellar height			
Postoperative ISI	1.2 (1.1–1.3)	1.1 (1.0–1.1)	<0.001^b^
ISI change (preop−postop)	0.1 ± 0.0	0.0 ± 0.0	<0.001^a^
Postoperative CDI	1.1 ± 0.2	1.1 ± 0.2	0.129^a^
CDI change (preop−postop)	0.0 (0.0–0.1)	0.0 (0.0–0.1)	0.869^b^
Postoperative BPR	0.9 ± 0.2	0.8 ± 0.2	0.361^a^
Postoperative patellar tilt (degrees)	13.6 ± 6.6	12.7 ± 6.6	0.508^a^
Functional outcomes at final follow-up			
Kujala score (0–100)	73.0 (58.0–80.0)	84.5 (76.0–90.0)	<0.001^b^
Lysholm score (0–100)	77.0 (62.0–86.0)	85.5 (74.2–92.0)	0.003^b^
VAS pain score (0–10)	2.0 ± 1.3	2.0 ± 1.2	0.740^a^
Range of motion (degrees)	113.0 (100.0–122.0)	132.5 (118.2–139.8)	<0.001^b^
Stability outcomes			
Redislocation, *n* (%)	12 (24.5)	4 (7.4)	0.034^c^
Subluxation, *n* (%)	17 (34.7)	8 (14.8)	0.034^c^
Poor outcome, *n* (%)	22 (44.9)	10 (18.5)	0.007^c^
Revision surgery, *n* (%)	5 (10.2)	2 (3.7)	0.253^d^
Postoperative positive J-sign, *n* (%)	26 (53.1)	17 (31.5)	0.044^c^
Postoperative positive apprehension, *n* (%)	21 (42.9)	10 (18.5)	0.013^c^
Return to sport			
Time to return to sport (months)	7.8 ± 2.4	6.9 ± 1.6	0.023^a^
Same level, *n* (%)	30 (61.2)	35 (64.8)	
Lower level, *n* (%)	8 (16.3)	10 (18.5)	
Not returned, *n* (%)	10 (20.4)	4 (7.4)	
Complications			
Wound infection, *n* (%)	0 (0.0)	0 (0.0)	1.000^d^
Arthrofibrosis, *n* (%)	2 (4.1)	2 (3.7)	1.000^d^
Hardware-related problem, *n* (%)	0 (0.0)	1 (1.9)	1.000^d^

Data are presented as mean ± SD for normally distributed continuous variables, median (Q1, Q3) for non-normally distributed continuous variables, and *n* (%) for categorical variables. ISI, Insall-Salvati index; CDI, Caton-Deschamps index; BPR, Blackburne-Peel ratio; ROM, range of motion; VAS, visual analog scale; RTS, return to sport.

a Independent samples *t*-test.

b Mann–Whitney *U* test.

c Chi-square test.

d Fisher's exact test.

Regarding patellar stability, the patella alta group had higher rates of redislocation (24.5% vs. 7.4%, *P* = 0.034), subluxation (34.7% vs. 14.8%, *P* = 0.034), and overall poor outcomes (44.9% vs. 18.5%, *P* = 0.007) compared to the normal group. Positive postoperative J-sign (53.1% vs. 31.5%, *P* = 0.044) and apprehension test (42.9% vs. 18.5%, *P* = 0.013) were also more frequent in the patella alta group. The revision rate was numerically higher in the patella alta group (10.2% vs. 3.7%) but did not reach statistical significance (*P* = 0.253). When comparing the magnitude of functional improvement, the median Kujala score gain (final minus preoperative) was 22.0 points (IQR 2.0–31.0) in the patella alta group and 27.0 points (IQR 18.2–35.8) in the normal group, with the between-group difference reaching statistical significance (*P* = 0.026). Postoperative ISI normalization within the patella alta group did not appear to affect clinical outcomes: the rate of poor outcomes among patients whose ISI normalized to ≤1.2 (45.8%) was similar to that among patients whose ISI remained elevated (44.0%).

### 3.4 Correlation between ISI and functional outcomes

As shown in Figure 3, preoperative ISI showed a significant negative correlation with final Kujala score (Spearman *ρ* = −0.457, *P* < 0.001). A weaker but significant negative correlation was also observed between preoperative ISI and final Lysholm score (*ρ* = −0.294, *P* = 0.003). No significant correlation was found between preoperative ISI and final VAS (*ρ* = −0.000, *P* = 0.996). In contrast, preoperative CDI showed no significant correlation with final Kujala score (*ρ* = −0.113, *P* = 0.254) or final Lysholm score (*ρ* = −0.025, *P* = 0.805). With respect to recurrent instability specifically, a higher preoperative ISI was associated with the composite endpoint of recurrent instability (redislocation or subluxation), with a logistic-regression odds ratio of 1.33 per 0.1-unit increase in ISI (95% CI 1.00–1.77, *P* = 0.048); the association with redislocation alone did not reach significance (odds ratio 1.25 per 0.1-unit increase, 95% CI 0.88–1.75, *P* = 0.209).

**Figure 3:**
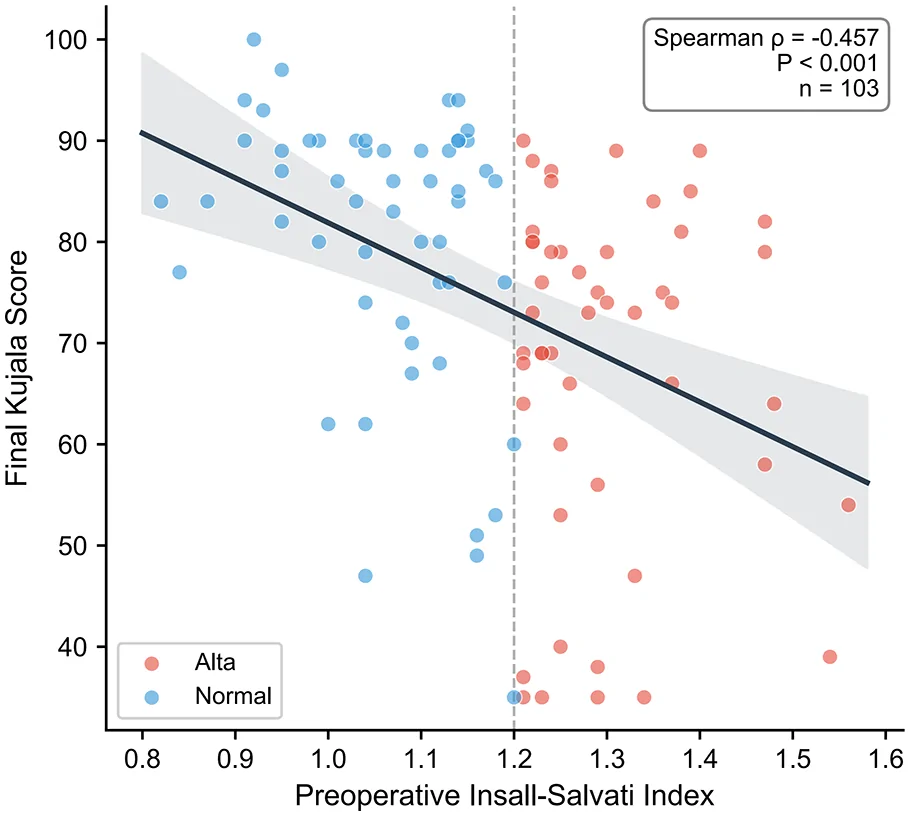
Correlation between preoperative ISI and final Kujala score. Scatter plot showing the relationship between preoperative Insall-Salvati index and final follow-up Kujala score for the entire cohort. The solid line represents the linear regression fit, and the shaded area represents the 95% confidence interval. The dashed vertical line marks the ISI = 1.2 threshold. ISI, Insall-Salvati index.

### 3.5 Independent predictors of poor outcome and anatomical risk-factor burden

In multivariable analysis adjusting for BMI, preoperative Lysholm score, high-grade trochlear dysplasia (Dejour type B or D), and TT-TG distance, patella alta remained independently associated with a poor outcome [adjusted odds ratio [OR] 3.83, 95% confidence interval [CI] 1.52–9.62, *P* = 0.004]; each 0.1-unit increase in preoperative ISI corresponded to an adjusted OR of 1.36 (95% CI 1.01–1.81, *P* = 0.040). In the corresponding linear model, higher preoperative ISI independently predicted a lower final Kujala score (*β* = −4.63 points per 0.1-unit increase, *P* < 0.001), whereas neither BMI (*P* = 0.36) nor preoperative Lysholm score (*P* > 0.99) was independently associated with outcome. In contrast, postoperative ISI was not independently associated with poor outcome (adjusted OR 1.26 per 0.1-unit increase, 95% CI 0.93–1.69, *P* = 0.13), and within the patella alta group the poor-outcome rate did not differ between patients whose ISI normalized and those in whom it remained elevated (45.8% vs. 44.0%, *P* = 1.00). With respect to the burden of coexisting anatomical risk factors, 13 of the 49 patella alta patients (26.5%) had isolated patella alta, whereas 36 (73.5%) had at least one additional risk factor. The poor-outcome rate rose progressively with the number of coexisting anatomical risk factors (0, 1, 2, 3, and 4 factors: 15.8%, 35.3%, 30.8%, 44.4%, and 50.0%, respectively), although this trend was not statistically significant (Cochran-Armitage *P* = 0.157).

### 3.6 Subgroup analyses by age and skeletal maturity

The adverse association between patella alta and outcome was more pronounced in younger and skeletally immature patients. Among patients ≤ 15 years (*n* = 76), the patella alta group had a higher rate of poor outcome than the normal group (51.4% vs. 14.6%, *P* = 0.001) and a lower median final Kujala score (74.0 vs. 85.0, *P* < 0.001); among those > 15 years (*n* = 27), the between-group difference in poor outcome was no longer significant (28.6% vs. 30.8%, *P* = 1.00), although the Kujala score remained lower in the patella alta group (*P* = 0.044). A similar pattern was observed by skeletal maturity: among patients with an open distal femoral physis (*n* = 67), the poor-outcome rate was 54.8% vs. 16.7% (*P* = 0.002), whereas among those with a closed physis (*n* = 36) the difference was not significant (27.8% vs. 22.2%, *P* = 1.00). The age-by-group interaction approached significance (*P* = 0.057).

## 4 Discussion

The present study evaluated the prognostic significance of preoperative ISI in children undergoing MPFL reconstruction for recurrent patellar instability. The results demonstrated that MPFL reconstruction effectively reduced ISI in both groups, with a significantly greater decrease observed in the patella alta group. Despite this radiographic improvement, patients with preoperative patella alta showed inferior functional outcomes, including lower Kujala and Lysholm scores, higher redislocation and subluxation rates, and increased positive postoperative J-sign and apprehension tests. Preoperative ISI correlated negatively with final Kujala score (*ρ* = −0.457), while the CDI failed to predict functional outcomes or demonstrate between-group differences in postoperative change. These findings reveal a clinically relevant dissociation between ISI and CDI in the pediatric patellofemoral instability population.

The observation that MPFL reconstruction decreases patellar height is consistent with previous reports. Fabricant et al. (33) demonstrated significant postoperative improvement in all three patellar height indices in 27 children after hamstring autograft MPFL reconstruction, with values returning to normal childhood ranges. A matched cohort of 103 skeletally immature patients, confirmed significant reductions in ISI, CDI, and Blackburne-Peel ratio following isolated MPFL reconstruction (34). Computational modeling has provided a biomechanical basis for this phenomenon, demonstrating that the restored medial restraint shifts the patella into a more distal tracking position during early knee flexion, and such reductions have been shown to remain stable at 5-year follow-up (35, 36). The underlying mechanism likely involves alteration of the patellar equilibrium position: by restoring the medial check-rein function, MPFL reconstruction counteracts the lateral and proximal forces that maintain the patella in a pathologically elevated position (35, 37). Nevertheless, the clinical significance of radiographic patellar height normalization appears limited. In the present cohort, 49.0% of patella alta patients achieved postoperative ISI within the normal range (≤1.2), yet their rate of poor outcomes (45.8%) was virtually identical to those whose ISI remained elevated (44.0%). A comparable observation has been reported in 283 adult patients, where CDI normalization occurred in 59.6% but had no measurable effect on Banff Patella Instability Instrument scores (38). These findings suggest that ISI improvement after MPFL reconstruction may represent a secondary biomechanical consequence of the procedure, not the primary mechanism through which clinical benefit is achieved. Consistent with this interpretation, neither postoperative ISI nor its normalization was independently associated with poor outcomes in the present multivariable analysis, whereas preoperative ISI was. Furthermore, patella alta may serve as a surrogate marker for a broader constellation of anatomic abnormalities, instead of functioning as an isolated causal factor.

Whether patella alta independently predicts outcomes after MPFL reconstruction remains a subject of debate. In a series of 211 reconstructions with a minimum 3-year follow-up, CDI ≥1.3 was identified as a strong risk factor for failure with an odds ratio of 4.9 (17). A recent meta-analysis pooling 807 patients found similar association, additionally identifying Dejour type B/D trochlear dysplasia (OR = 3.28) as an independent predictor of recurrent instability (39). In the pediatric population, trochlear dysplasia and high-grade J-sign have been reported as the primary risk factors for MPFL reconstruction failure (40). A failure rate of 24.1% has been observed in skeletally immature patients when bony risk factors were not simultaneously corrected, with comparable recurrence patterns documented in adolescent allograft cohorts (9, 41). In contrast, favorable 5-year outcomes have been reported in patients treated with isolated MPFL reconstruction regardless of patellar height and TT-TG distance, with only 5% experiencing recurrent instability (42). A pooled analysis has similarly shown that neither patella alta nor increased TT-TG adversely affected patient-reported outcomes (43). The present study offers a potential explanation for these conflicting results: the predictive capacity of patellar height depends critically on the index used. ISI demonstrated significant correlations with functional outcomes (*ρ* = −0.457 for Kujala), while CDI showed none (*ρ* = −0.113). Studies supporting a negative prognostic effect have predominantly relied on CDI in adult populations, whereas those finding no effect have employed varied indices across mixed populations (17, 42). This discrepancy suggests that the choice of measurement index represents an underrecognized source of heterogeneity in the existing literature. Although preoperative ISI remained independently associated with a poor outcome after adjustment for BMI, preoperative Lysholm score, and other anatomical risk factors, the effect sizes were modest and were derived from a single-center cohort; the apparent superiority of the ISI over the CDI should therefore be regarded as hypothesis-generating rather than definitive.

The agreement between ISI and CDI in classifying patellar height was essentially absent in the present cohort (Cohen's kappa = −0.017), with only 11 of 49 ISI-classified patella alta patients simultaneously meeting CDI criteria. This observation is not confined to pediatric populations; a kappa of only 0.01 between ISI and CDI has been reported in healthy adult knees, confirming that these indices classify the same anatomic morphology differently at a fundamental level (44). The discordance is likely amplified in children because skeletal maturation affects measurement reference points. In healthy children, CDI values have been shown to decrease with age as patellar ossification progresses, indicating that age-specific normative values are essential when applying CDI in the pediatric population. Subsequent studies have confirmed that patellar height classification in the pediatric knee varies significantly depending on the measurement method, age group, and sex (19, 20). These findings carry implications for index selection: CDI references the inferior articular surface of the patella relative to the tibial plateau, landmarks that may undergo considerable morphological change during skeletal growth. ISI measures the ratio of patellar tendon length to patellar diagonal length, anatomic structures that are more consistently identifiable regardless of skeletal maturity.

The absence of between-group differences in postoperative VAS pain scores, despite significant differences in Kujala and Lysholm scores, may be attributed to a floor effect, as both groups achieved median VAS values of 1.8, potentially leaving insufficient range to detect meaningful differences. The Kujala and Lysholm instruments incorporate functional activities such as squatting, stair climbing, and running, and therefore captured residual functional limitations that a unidimensional pain scale could not. Data from a multicenter study have shown that the Banff Patella Instability Instrument 2.0 is the only patient-reported outcome measure without ceiling effects following patellofemoral stabilization surgery in adolescents, outperforming the Kujala score in sensitivity to change (45). This scale in future pediatric patellofemoral instability research may provide the discriminatory power needed to detect outcome differences between subgroups that conventional scales fail to capture.

Several limitations should be acknowledged. The retrospective design cannot establish causality, and selection bias inherent to single-center data may limit generalizability. The sample of 103 patients, while comparable to the largest pediatric series on this topic, constrains statistical power for rare events such as revision surgery, where the observed difference (10.2% vs. 3.7%) did not reach statistical significance. The ISI threshold of 1.2 used for group classification derives from adult normative data, and although existing pediatric studies have reported similar normal ranges, a child-specific cutoff validated through receiver operating characteristic analysis would strengthen classification accuracy. The minimum follow-up of 6 months may not capture late recurrences, as patellar instability can recur years after surgery. Additionally, VAS and Kujala scores have recognized limitations in this population, and the retrospective nature of functional score collection introduces potential recall bias. The subgroup analyses by age and skeletal maturity were exploratory and, particularly in the older and skeletally mature subgroups, were underpowered; the suggestion that the adverse effect of patella alta is more pronounced in younger patients (age-by-group interaction *P* = 0.057) should be interpreted with caution and confirmed in larger cohorts. The count of coexisting anatomical risk factors, moreover, relied on pragmatic thresholds that are not universally standardized. Future prospective multicenter studies with longer follow-up and incorporation of disease-specific instruments are needed to validate and extend these findings.

## 5 Conclusions

This retrospective study found that preoperative patella alta, as measured by the Insall-Salvati index, was associated with inferior functional scores, reduced range of motion, and higher rates of recurrent instability following MPFL reconstruction in pediatric patients. Although MPFL reconstruction was associated with a reduction in patellar height in both groups, postoperative normalization of the ISI did not appear to translate into improved clinical outcomes, suggesting that patella alta may represent a marker of a broader constellation of anatomic risk factors rather than an isolated modifiable variable. Notably, the ISI was more strongly associated with clinical outcomes than the Caton-Deschamps index in this cohort, with poor agreement between the two indices in classifying patellar height. Because these observations derive from a single-center, retrospective cohort with modest effect sizes, they should be interpreted as hypothesis-generating and warrant prospective, multicenter validation. These findings suggest that preoperative ISI assessment may aid in patient counseling and risk stratification when planning isolated MPFL reconstruction in children with recurrent patellar instability.

## Data Availability

The original contributions presented in the study are included in the article/Supplementary Material, further inquiries can be directed to the corresponding authors.
